# Antiproliferative Activity and Potential Mechanism of Marine-Sourced Streptoglutarimide H against Lung Cancer Cells

**DOI:** 10.3390/md19020079

**Published:** 2021-01-31

**Authors:** Hengju Ge, Di Zhang, Muran Shi, Xiaoyuan Lian, Zhizhen Zhang

**Affiliations:** 1 Zhoushan Campus, Ocean College, Zhejiang University, Zhoushan 316021, China; hjge@zju.edu.cn (H.G.); dizhang@jou.edu.cn (D.Z.); 2 College of Pharmaceutical Sciences, Zhejiang University, Hangzhou 310058, China; mrshi@zju.edu.cn; 3 Jiangsu Key Laboratory of Marine Pharmaceutical Compound Screening, Jiangsu Ocean University, Lianyungang 222005, China

**Keywords:** Streptoglutarimide H, lung cancer cells, antiproliferative activity, cell cycle block, glycolysis, USP28, c-Myc

## Abstract

In 2019, streptoglutarimide H (SGH) was characterized as a new glutarimide from the secondary metabolites produced by a marine-derived actinomycete *Streptomyces* sp. ZZ741 and shown to have in vitro antiglioma activity. However, the antiproliferative activity and potential mechanism of SGH against lung cancer cells have not yet been characterized. This study demonstrated that SGH significantly inhibited the proliferation of different lung cancer cells. In terms of mechanism of action, SGH downregulated cell cycle- and nucleotide synthesis-related proteins to block cell cycle at G0/G1 phase, reduced the expression levels of glycolytic metabolic enzymes to inhibit glycolysis, and downregulated the important cancer transcription factor c-Myc and the therapeutic target deubiquitinase USP28. Potent anticancer activity and multiple mechanisms indicated SGH to be a novel antitumor compound against lung cancer cells.

## 1. Introduction

Lung cancer is the world’s leading cause of cancer death. Non-small cell lung cancer (NSCLC) is the most common type of lung cancer and account for approximately 80–90% of all lung cancers [1,2]. Platinum-based doublet chemotherapy was considered the good standard for the treatment of advanced NSCLC without gene mutation and the discovery of targetable oncogenic mutations revolutionized treatment choices for NSCLC [3]. Epidermal growth factor receptor (EGFR) mutations are usually observed in patients with NSCLC. First-generation (gefitinib, erlotinib) and second-generation (afatinib, dacomitinib) EGFR-tyrosine kinase inhibitors (TKIs) with platinum-based doublet chemotherapy established first- and second-generation EGFR-TKIs as the standard-of-care for patients with EGFR-mutated advanced NSCLC. Furthermore, osimertinib, a third-generation EGFR-TKI that is highly selective for the EGFR-activating mutation and EGFR T790M resistance mutation in NSCLC patients, has shown superior efficacy versus first-generation EGFR-TKIs [3]. However, NSCLC patients treated with first- or second-generation EGFR-TKIs, even osimertinib, inevitably developed resistance [3,4,5]. The overall cure and survival rates for NSCLC by the use of small molecule EGFR-TKIs and immunotherapy remain low, particularly in metastatic disease [6]. Therefore, there is a continued need to discover and develop new drugs with novel mechanisms of action for the treatment of NSCLC.

Ubiquitination has shown to play multiple roles in cancer-related pathways and ubiquitin E3 ligases are central to the ubiquitin-conjugation system and regulate numerous cellular processes, including metabolism, cell cycle progression, and transcription [7,8]. By counteracting the activities of the E3 ligases, deubiquitylating enzymes have been suggested as an important mechanism to regulate the ubiquitination process in cancer. USP28 (ubiquitin-specific proteases 28) is a deubiquitinase that has been recommended as a promising diagnostic marker and therapeutic target for NSCLC [8,9].

Marine natural products are important resources for the discovery and development of new anticancer drugs or drug leads. Since the approval of the first marine anticancer drug cytarabine, the marine-derived anticancer pharmaceutical pipeline has produced four approved drugs (cytarabine, trabectedin, eribulin, and brentuximab vedotin) and eighteen drug candidates, such as salinosporamide A and plinabulin, in clinical trials [10]. 

Streptoglutarimide H (SGH, Figure S1) was initially isolated and identified as a new compound from a culture of the marine-derived actinomycete *Streptomyces* sp. ZZ741 in rice medium, with potent in vitro antiproliferative activity against human glioma U251 and U87MG cells [11]. However, the activity and mechanism of SGH against lung cancer cells have not yet been characterized. The aim of this study is to investigate the antiproliferative activity and potential mechanism of streptoglutarimide H against lung cancer cells.

## 2. Results 

### 2.1. Streptoglutarimide H (SGH) Inhibited the Proliferation and Colony Formation of Lung Cancer Cells

**Figure 1:**
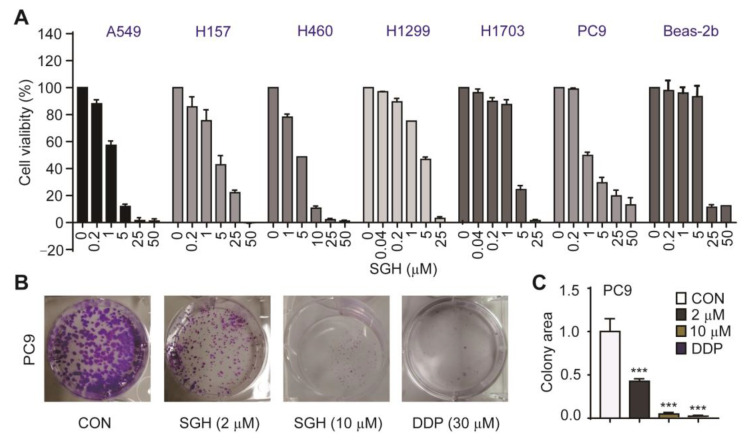
Streptoglutarimide H (SGH) inhibited the proliferation of lung cancer cells and normal lung Beas-2b cells. (**A**) Cell viability of different lung cancer cells and Beas-2b cells treated with different concentrations of SGH for 72 h. (**B**) Cell colony of PC9 cells treated with SGH (2, 10 µM) or DDP (30 µM) for 2 weeks. (**C**) Quantitative results of the cell colony in Figure 1B. Data are presented as the mean ± SD (*n* = 3, three independent experiments), *** *p* < 0.001 (vs. CON) by one-way ANOVA.

The antiproliferative activity of SGH towards human lung cancer A549, H157, H460, H1299, H1703, and PC9 cells were determined by sulforhodamine B (SRB) assay. Two drugs cisplatin (DDP) and afatinib were used as positive controls. The results (Figure 1A, Table S1) indicated that SGH had potent activity in inhibiting the proliferation of all six tested cell lines with IC_50_ values of 1.69–5.24 µM. The control drug DDP showed antiproliferative activity with IC_50_ values of 0.71–5.97 µM, while imatinib showed high variation in sensitivity to lung cancer cells with IC_50_ values of 0.003 µM for PC9 cells and 0.32–3.94 µM for others. The cytotoxicity (CC_50_) of SGH, DDP, and afatinib towards normal lung Beas-2b cells was also investigated (Figure 1A and Figure S2, Table S1). The CC_50_ values against Beas-2b cells were 17.32 µM for SGH, 38.80 µM for DDP, and 2.64 µM for afatinib. Further investigation confirmed that SGH at 2 µM for PC9 cells and 5 µM for H157 cells significantly reduced the cell colony formation (*** *p* < 0.001, Figure 1B,C and Figure S3). The data indicated that SGH had potent in vitro antiproliferative activity against lung cancer cells.

### 2.2. Streptoglutarimide H (SGH) Blocked Cell Cycle at G0/G1 Phase by Downregulating the Cell Cycle- and Nucleotide Synthesis-related Proteins in Lung Cancer Cells

**Figure 2:**
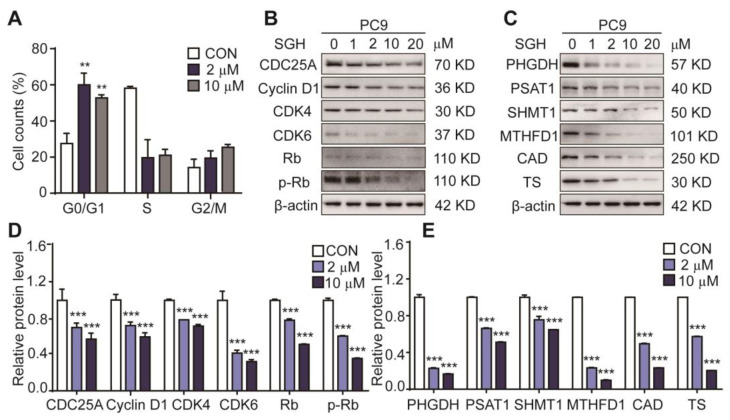
Streptoglutarimide H (SGH) arrested cell cycle at G0/G1 phase and downregulated the cell cycle- and nucleotide synthesis-related proteins in lung cancer PC9 cells. (**A**) Percentage of cells at each stage of the cell cycle in PC9 cells treated with SGH (2, 10 µM) for 24 h. (**B**,**C**) Expressions of the cell cycle- and nucleotide synthesis-related proteins in PC9 cells treated with different concentrations of SGH for 24 h. (**D**,**E**) Quantitative results of the protein levels of cell cycle- and nucleotide synthesis-related regulators in Figure 2B,C. Data are presented as the mean ± SD (*n* = 3, three independent experiments), ** *p* < 0.01, *** *p* < 0.001 (vs. CON) by one-way ANOVA.

To explore whether the antiproliferative activity of SGH is related to cell cycle arrest, the effects of SGH on cell cycle and cell cycle regulators were investigated. As shown in Figure 2A, Figures S4, S5A and Table S2, the percentage of cells in the G0/G1 phase of cell cycle increased 29.32 % and 25.18 % in PC9 cells treated with SGH at 2 µM and 10 µM for 24 h, respectively, when compared to the control group (CON). Similar results were also obtained in H157 cells. The data suggested that SGH blocked PC9 and H157 cells at the G0/G1 phase. It is well known that specific cyclins, cyclin-dependent kinases (CDKs), retinoblastoma (Rb), and phosphorylated retinoblastoma (p-Rb) control the progression of cell cycle in cancer cells [12,13]. Western blot analysis indicated that SGH at 2 µM for PC9 cells and 5 µM for H157 cells downregulated all cell cycle regulators of CDC25A, CDK4, CDK6, cyclin D1, Rb, and p-Rb (*** *p* < 0.001, Figure 2B,D and Figure S5B,D). 

The G0/G1 phase of cell cycle is the early stage of DNA synthesis. Thus, we also assessed the effect of SGH on the expression of nucleotide synthesis-related proteins, including PHGDH and PSAT1 (two catalytic enzymes in the serine biosynthesis pathway), SHMT1 and MTHFD1 (two catalytic enzymes in the one-carbon metabolism pathway), CAD and TS (two catalytic enzymes in the nucleic acid synthesis pathway). The results (Figure 2C,E and Figure S5C,E) show that SGH at 2 µM for PC9 cells and 5 µM for H157 cells downregulated all six tested proteins after a 24 h treatment (*** *p* < 0.001). These data suggested that SGH can regulate cell cycle- and nucleotide synthesis-related proteins, thereby arrested cell cycle at G0/G1 phase in lung cancer cells.

### 2.3. Streptoglutarimide H (SGH) Inhibited Glycolysis in Lung Cancer Cells

**Figure 3:**
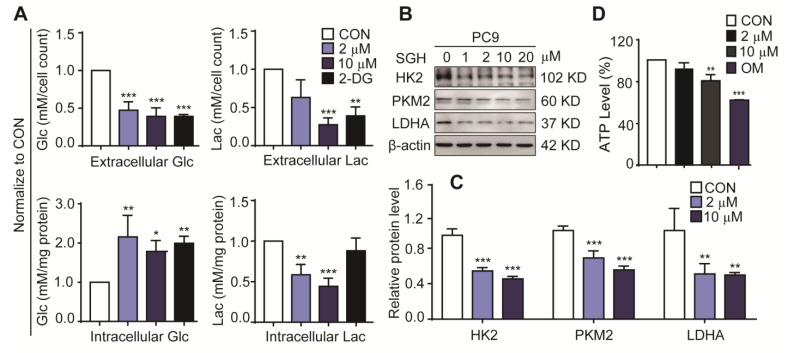
Streptoglutarimide H (SGH) inhibited glycolysis in lung cancer PC9 cells. (**A**) Extracellular and intracellular glucose (Glc) and lactate (Lac) levels in PC9 cells treated with SGH (2, 10 µM) and 2-DG (0.4 mM) for 24 h. (**B**) Levels of glycolytic regulators HK2, PKM2, and LDHA in PC9 cells treated with different concentrations of SGH. (**C**) Quantitative results of HK2, PKM2, and LDHA levels in Figure 3B. (D) ATP level in PC9 cells treated with SGH (2, 10 µM) and oligomycin (OM, 10 µM) for 24 h. Data are presented as the mean ± SD (*n* = 3, three independent experiments), * *p* < 0.05, ** *p* < 0.01, *** *p* < 0.001 (vs. CON) by one-way ANOVA.

High consumption of glucose and mass production of lactate are important characteristics of enhanced glycolysis in cancer cells [14,15,16,17]. The reduction of glucose consumption and lactate production in tumors is related to the regulation of tumor glycolysis and has antitumor activity [18,19]. Several important glycolytic regulators including hexokinase 2 (HK2) [14,20], pyruvate kinase M2 (PKM2) [14,21], and lactate dehydrogenase A (LDHA) [16,22], have been proved to be upregulated in the cancer cells. These specific tumor regulators are important targets of anticancer drugs [14,15,22]. Therefore, the effects of SGH on the glucose consumption, lactate production, and expression levels of the glycolytic regulators HK2, PKM2, and LDHA in lung cancer cells were investigated. 2-deoxyglucose (2-DG) (a hexokinase 2 inhibitor) was used as a positive control. As shown in Figure 3A and Figure S6A, a 24 h treatment of SGH at 2 µM for PC9 cells and 5 µM for H157 cells decreased the extracellular glucose consumption and lactate release (** *p* < 0.01 or *** *p* < 0.001). At the same time, increased intracellular glucose and decreased intracellular lactate were also observed in both PC9 and H157 cells treated with SGH (2 or 5 µM) for 24 h (** *p* < 0.01 or *** *p* < 0.001). SGH (2 or 5 µM) also downregulated three important glycolytic regulators, HK2, PKM2, and LDHA, in both PC9 and H157 cells (** *p* < 0.01 or *** *p* < 0.001) (Figure 3B,C and Figure S6B,C). In addition, SGH (10 µM or 15 µM) reduced ATP production in both PC9 and H157 cells (** *p* < 0.01 or *** *p* < 0.001, Figure 3D and Figure S6D). All data together suggested that the antiproliferative activity of SGH was related to the inhibition of the enhanced glycolysis in lung cancer cells.

### 2.4. Streptoglutarimide H (SGH) Downregulated Deubiquitinase USP28 and Cancer Transcription Factor c-Myc

**Figure 4:**
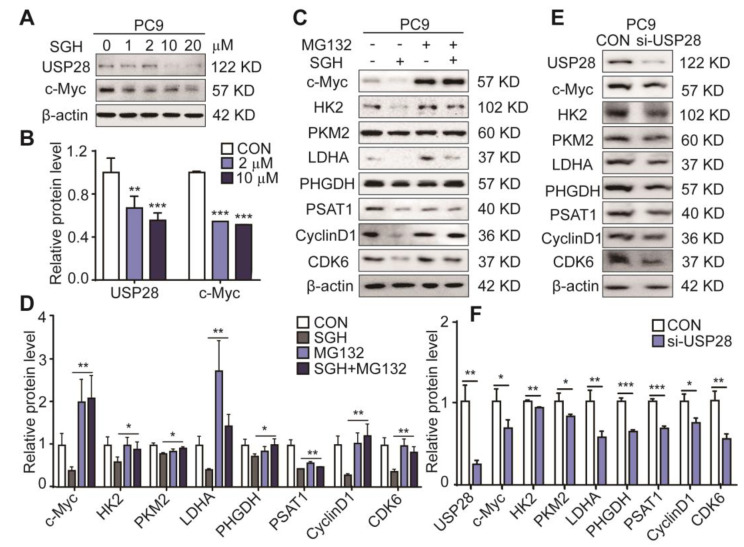
Streptoglutarimide H (SGH) downregulated USP28 and c-Myc, proteasome inhibitor MG132 reversed the downregulation of related proteins induced by SGH, and si-USP28 downregulated the cell cycle-, glycolysis-, and nucleotide synthesis-related regulators in PC9 cells. (**A**) Protein levels of USP28 and c-Myc in PC9 cells treated with different concentrations of SGH for 24 h. (**B**) Quantitative result of USP28 and c-Myc levels in Figure 4A. (**C**) Expressions of c-Myc, HK2, PKM2, LDHA, PHGDH, PSAT1, CDK6, and cyclin D1 in PC9 cells preincubated with MG132 (10 µM) for 2 h and then SGH (10 µM) for 6 h. (**D**) Quantitative results of the protein levels in Figure 4C. (**E**) Expressions of USP28, c-Myc, HK2, PKM2, LDHA, PHGDH, PSAT1, CDK6, and Cyclin D1 in PC9 cells after the application of 48 h with 20 pmol siRNA. (**F**) Quantitative results of the protein levels in Figure 4E. Data are presented as the mean ± SD (*n* = 3, three independent experiments), * *p* < 0.05, ** *p* < 0.01, *** *p* < 0.001 (SGH + MG132 vs. SGH or si-USP28 vs. CON) by students *t*-test.

The deubiquitinase USP28 has been recognized as a therapeutic target for NSCLC. Overexpressing USP28 was found in NSCLC tumors and enhanced NSCLC cell proliferation, and low survival was related to high USP28 levels in patients. Downregulating USP28 expression can inhibit the proliferation and growth of lung cancer cells [8,23]. USP28 is also reported to control c-Myc driving cancer cells [8] and the stability of c-Myc [24,25]. Therefore, the effect of SGH on the expression levels of USP28 and c-Myc was determined first. A 24 h treatment of SGH at 2 µM for PC9 cells and 5 µM for H157 cells downregulated USP28 and c-Myc expressions (** *p* < 0.01 or *** *p* < 0.001, Figure 4A,B and Figure S7A,B). Then, proteasome inhibitor MG132 was used to inhibit the ubiquitin-dependent protein degradation, the downregulation of c-Myc, HK2, PKM2, LDHA, PHGDH, PSAT1, cyclin D1, and CDK6 in both PC9 and H157 cells by SGH was reversed by MG132 (10 µM) (* *p* < 0.05 or ** *p* < 0.01, Figure 4C,D and Figure S7C,D). Next, lung cancer PC9 and H157 cells were transfected with siRNA-USP28 (si-USP28). It was found that the expression levels of the nine tested proteins (c-Myc, USP28, HK2, PKM2, LDHA, PHGDH, PSAT1, cyclin D1, and CDK6) in both PC9 and H157 cells were decreased after the application of si-USP28 (20 pmol) for 48 h (* *p* < 0.05, ** *p* < 0.01 or *** *p* < 0.001, Figure 4E,F and Figure S7E,F). The effects of SGH and si-USP28 on the downregulation of these proteins were similar, although they showed different effects in degree. All data together indicated that the downregulation of c-Myc, HK2, PKM2, LDHA, PHGDH, PSAT1, cyclin D1, and CDK6 in lung cancer cells was related to the ubiquitin-protein degradation pathway, which was the result that the USP28 was inhibited by SGH.

## 3. Discussion

This study demonstrated that streptoglutarimide H (SGH), a newly discovered marine natural product, has potent activity in suppressing the proliferation of different lung cancer cells. SGH downregulated several cell cycle- and nucleotide synthesis-related proteins, leading to the cell cycle arrest at G0/G1 phase; SGH also decreased the expressions of three key glycolytic regulators, resulting in the inhibition of the enhanced glycolysis in cancer cells.

Interestingly, SGH also downregulated the levels of USP28 and c-Myc. The transcription factor c-Myc plays an important role in the regulation of cell cycle through modulating cell cycle controllers, such as CDC25A, CDK4, CDK6, Rb, and p-Rb in cancer cells [12,13,26]. c-Myc has been demonstrated to increase the expressions of many tumor metabolic enzymes including glycolytic regulators HK2, PKM2, and LDHA to enhance the glycolysis in tumor cells [18,27]. Although c-Myc inhibition would be a powerful approach for the treatment of cancers, strategies to directly target c-Myc have not yet been achieved because c-Myc is easy to be degraded through the ubiquitin-proteasome pathway [25,28]. The deubiquitinase USP28 was shown to bind c-Myc through an interaction with FBW7 ubiquitin ligase, a tumor suppressor, leading to c-Myc stabilization and tumor cell proliferation [8]. Based on all data obtained from this study, we proposed a hypothesis that the anticancer activity of SGH may be the result of c-Myc destabilization through the downregulation of USP28, resulting in the downregulation of cell cycle-, nucleotide synthesis-, and glycolysis-related regulators to arrest cell cycle and inhibit glycolysis in lung cancer cells. This proposed mechanism needs to be further studied.

The USP28 deubiquitinase has been recognized as a promising therapeutic target for cancer [8,29]. Benzylaminoethanols (AZ1–AZ4) were the first reported compounds to directly and effectively target USP28 [29]. More recently, a few synthesized USP28 inhibitors were also reported [30,31]. To the best of our knowledge, streptoglutarimide H is the first USP28 inhibitor found from natural resource to display potent antiproliferative activity against lung cancer cells with unique mechanism of action.

SGH is an isomer of known streptovitacin A and both were isolated from the culture of *Streptomyces* sp. ZZ741 [11]. The known streptovitacin A was previously reported to have translation inhibitory activity [32], antitumor activity against lymphoma [33] and glioma [11], and antifungal activity [34]. It was noted that streptovitacin A showed 17–30 times more potent antiglioma activity than SGH [11]. Therefore, the structure–activity relationship of this type of compounds is worthwhile to be further investigated.

## 4. Materials and Methods

### 4.1. Lung Cancer Cells and Cell Culture

Human lung cancer A549 (CBP60084), H157 (CBP60952), H460 (CBP60138), H1299 (CBP60053), H1703 (CBP60053), and PC9 (CBP60178) cell lines and normal lung Beas-2b cells were obtained from CoBioer (Nanjing, China). The H157, PC9, H460, H1703, H1299, and Beas-2b cells were cultured in RPMI-1640 medium, and A549 cells in F12K medium. The media were supplemented with 10% fetal bovine serum (FBS) and 1% penicillin-streptomycin. All cells were maintained in a 5% CO_2_ humidified incubator at 37 °C and the cultured cells after the third generation were used for the experiments.

### 4.2. Agents

Streptoglutarimide H (SGH, purity over 98%) was isolated and purified from a culture of marine-derived actinomycete *Streptomyces* sp. ZZ741 in rice medium [11]. Afatinib was obtained from Aladdin (Shanghai, China), cisplatin (DDP) from Hansoh Pharma (Jiangsu, China), 2-deoxyglucose (2-DG) from Tokyo Chemical Industry (Shanghai, China), oligomycin (OM) and MG132 from MedChemExpress (Princeton, NJ, USA), crystal violet and propidium iodide (PI) from Solarbio (Beijing, China), sulforhodamine B (SRB) and RNase A from Sigma-Aldrich (St. Louis, MO, USA). Glucose assay kit was purchased from Rongsheng (Shanghai, China), lactate assay kit from Jiancheng (Nanjing, China), ATP assay kit from Beyotime (Shanghai, China). All the antibodies used in this study are listed in Table S3 (Supplementary Material) .

### 4.3. Sulforhodamine B (SRB) Assay

SRB assay [35,36] was used to determine the antiproliferative activity of SGH. Cisplatin (DDP) and afatinib were used as a positive control. Cell viability (%) = T_OD_/C_OD_ × 100%, where T_OD_ is the optical density (OD) value of tested compound and C_OD_ is the OD value of blank control (0.3% DMSO in water). IC_50_ values obtained by using GraphPad software are presented as the mean SD (*n* = 3, three independent experiments).

### 4.4. Colony Formation Assay

Lung cancer PC9 and H157 cells (500 cells/well) were treated with different concentrations (2–15 µM) of SGH or positive control drug cisplatin (DDP, 30 µM) [37] and inoculated in 6-well plates for 2 weeks. The treated-cells were washed with phosphate-buffered saline (PBS), fixed with 10% paraformaldehyde (PFA), and then stained with a solution of 0.1% crystal violet solution. After that, cell colonies were photographed, and area of each cell colony and total areas of all cell colonies were calculated by using Image J software (Version No. 1.4.3.67, National Institutes of Health, Bethesda, Maryland, USA). The SGH and DDP values were normalized to the control values and data are presented as the mean ± SD (*n* = 3, three independent experiments).

### 4.5. Cell Cycle Analysis

Cell cycle perturbations arrested by SGH were analyzed by propidium iodide (PI) DNA staining using flow cytometry. DDP was used as a positive control. PC9 and H157 cells at a density of 6 × 10^4^ per well were cultured in 6-well plates for 24 h and then treated with the SGH (2, 10 µM for PC9 cells, 5, 15 µM for H157 cells) or DDP (30 µM) for 24 h. After the treatment, cells were collected and then fixed with cold 70% ethanol at 4 °C overnight. The fixed cells were rinsed with cold PBS twice, resuspended in 500 µL PBS with 15 µL RNase A (50 µg/mL), incubated at 37 °C for 30 min, and then stained with 3 µL PI (10 µg/mL) at 4 °C for 5 min in dark. Immediately, cell cycle distribution was determined by flow cytometry (Cytomics^TM^ FC500 Flow Cytometry).

### 4.6. Cell Transfection

Lung cancer PC9 and H157 cells were cultured with RPMI-1640 medium supplemented with 10% FBS and 1% penicillin-streptomycin at 37 °C in a 5% CO_2_ humidified incubator. The logarithmic growth phase cells were washed with PBS, digested with trypsin (0.25%, Biosharp), and then planted into a 6-well plate. When cell density in the plate reached 70–80%, the cells were transfected with siRNAs (siRNA negative control: 5’-UUCUCCGAACGUGUCACGUTT-3’; siRNA-USP28: 5’-CCUUACUCAUGAUAACA AATT-3’) using Hieff Trans^TM^ Liposomal Transfection Reagent (Yeasen, Shanghai, China). The cells transfected with si-USP28 were harvested for protein extraction after the transfection of 48 h. The siRNA targeting USP28 was synthesized by Obio Technology (Shanghai, China). 

### 4.7. Measure of Glucose, Lactate, and ATP

By following the manufacturer’s instructions, the glucose assay kit, lactate assay kit, and ATP assay kit were used to determine the levels of glucose, lactate, and ATP in PC9 and H157 cells treated by different concentrations of SGH or positive control agents 2-deoxyglucose (2-DG, 0.4 mM) and oligomycin (OM, 10 µM) for 24 h. The levels of glucose and lactate in SGH and 2-DG groups were normalized to those in control (CON) group, and before that, the extracellular and intracellular glucose and lactate levels were respectively normalized by cell count and mg protein.

### 4.8. Western Blot Analysis

Western blot (WB) analysis was used to determine the expressions of the examined proteins. The detailed procedure, including protein sample preparation, determination of protein concentration, and western blot analysis, was described in the previous publication [38]. The antibodies used for WB analysis are listed in Table S3. β-actin was used as a loading control. Band intensities were quantified by using Image J software and data are presented as the mean ± SD (*n* = 3, three independent experiments).

### 4.9. Statistical Analysis

All data are presented as the mean ± SD (*n* = 3, three biological replicates from three independent experiments). GraphPad Prism 7.0 (GraphPad Software, San Diego, CA, USA) was used for statistical analysis. Comparisons between two groups were carried out with two-tailed students *t*-test. Variances between more than two groups were analyzed with one-way ANOVA. The *p* value < 0.05 was considered to indicate the statistical significance.

## Data Availability

Data is contained within the article or supplementary material.

## Supplementary Materials

The following are available online at https://www.mdpi.com/1660-3397/19/2/79/s1, Figure S1: Structure of streptoglutarimide H (SGH); Figure S2: Positive control drugs cisplatin (DDP) and afatinib inhibited the proliferation of lung cancer cells and normal lung Beas-2b cells; Figure S3: Streptoglutarimide H (SGH) and cisplatin (DDP) inhibited the colony formation of lung cancer H157 cells; Figure S4: Streptoglutarimide H (SGH) and cisplatin (DDP) arrested cell cycle at G0/G1 and S phases, respectively, in lung cancer PC9 and H157 cells; Figure S5: Streptoglutarimide H (SGH) arrested cell cycle at G0/G1 phase and downregulated the cell cycle- and nucleotide synthesis-related proteins in lung cancer H157 cells; Figure S6: Streptoglutarimide H (SGH) inhibited glycolysis in lung cancer H157 cells; Figure S7: Streptoglutarimide H (SGH) downregulated USP28 and c-Myc, proteasome inhibitor MG132 reversed the downregulation of related proteins induced by SGH, and si-USP28 downregulated the cell cycle-, glycolysis-, and nucleotide synthesis-related regulators in H157 cells; Table S1: Antiproliferative activity of streptoglutarimide H (SGH) and positive control drugs cisplatin (DDP) and afatinib against lung cancer cells and normal lung Beas-2b cells; Table S2: Percentage (%) of cells at each stage of the cell cycle in PC9 and H157 cells treated with streptoglutarimide H (SGH) and positive control drugs cisplatin (DDP); Table S3: Lists of antibodies used for western blot analysis.
