# Supplementary material for: Antiproliferative Activity and Potential Mechanism of Marine-Sourced Streptoglutarimide H against Lung Cancer Cells
Source: Mar Drugs. 2021 Jan 31;19(2):79. doi: 10.3390/md19020079 (PMC7911229; doi:10.3390/md19020079)
Supplement: Supplementary file 1 [file marinedrugs-19-00079-s001.pdf]

*SUPPLEMENTARY MATERIALS FOR*

# Antiproliferative activity and potential mechanism of marine-sourced streptoglutarimide H against lung cancer cells

Hengju Ge[1], Di Zhang[1,3], Muran Shi[2], Xiaoyuan Lian[2,] * and Zhizhen Zhang[1,] *

[1] Ocean College, Zhoushan Campus, Zhejiang University, Zhoushan 316021, China; hjge@zju.edu.cn (H.G.);

[2] College of Pharmaceutical Sciences, Zhejiang University, Hangzhou 310058, China; mrshi@zju.edu.cn (M.S.);

[3] Jiangsu Key Laboratory of Marine Pharmaceutical Compound Screening, Jiangsu Ocean University, Lianyungang, 222005, China; dizhang@jou.edu.cn (D.Z.)

[*] Correspondence: xylian@zju.edu.cn (X.L.); zzhang88@zju.edu.cn (Z.Z.); Tel.: +86-13575476388 (X.L.); +86-13675859706 (Z.Z.)

**Table S1.** Antiproliferative activity of streptoglutarimide H (SGH) and positive control drugs cisplatin (DDP) and afatinib against lung cancer cells and normal lung Beas-2b cells ($IC_{50}$: $\mu$M, mean ± SD, *n* = 3)

| Cells | A549 | H157 | H460 | H1299 | H1703 | PC9 | Beas-2b |
|---|---|---|---|---|---|---|---|
| SGH | 3.14 ± 0.39 | 5.24 ± 0.17 | 4.16 ± 0.39 | 2.98 ± 0.36 | 3.39 ± 0.14 | 1.69 ± 0.18 | 17.32 ± 1.24 |
| DDP | 1.52 ± 0.00 | 5.15 ± 0.16 | 1.13 ± 0.02 | 3.64 ± 0.01 | 5.97 ± 0.36 | 0.71 ± 0.01 | 38.80 ± 5.64 |
| Afatinib | 3.94 ± 0.40 | 0.32 ± 0.04 | 2.25 ± 0.14 | 1.08 ± 0.13 | 1.94 ± 0.21 | 0.003 ± 0.00 | 2.64 ± 0.23 |

**Table S2**. Percentage (%) of cells at each stage of the cell cycle in PC9 and H157 cells treated with streptoglutarimide H (SGH) and positive control drugs cisplatin (DDP) (mean ± SD, *n* = 3)

| Compound | PC9 | | | Compound | H157 | | |
|---|---|---|---|---|---|---|---|
| | G0/G1 | S | G2/M | | G0/G1 | S | G2/M |
| CON | 27.56 ± 4.58 | 58.20 ± 0.78 | 14.24 ± 3.78 | CON | 51.37 ± 6.70 | 33.97 ± 4.35 | 14.66 ± 5.68 |
| SGH (2 $\mu$M) | 56.88 ± 5.49 | 22.13 ± 6.65 | 20.99 ± 4.08 | SGH (5 $\mu$M) | 69.18 ± 5.59 | 22.50 ± 4.00 | 8.32 ± 3.70 |
| SGH–CON | +29.32 | – 36.07 | + 6.75 | SGH–CON | + 17.81 | – 11.47 | – 6.34 |
| SGH (10 $\mu$M) | 52.74 ± 1.60 | 21.00 ± 3.25 | 26.26 ± 1.60 | SGH (15 $\mu$M) | 69.78 ± 6.03 | 18.30 ± 2.84 | 11.92 ± 3.72 |
| SGH–CON | + 25.18 | – 37.20 | + 12.02 | SGH–CON | + 18.41 | – 15.67 | – 2.74 |
| DDP (30 $\mu$M) | 9.46 ± 1.78 | 76.85 ± 5.85 | 13.69 ± 7.64 | DDP (30 $\mu$M) | 34.47 ± 6.55 | 43.35 ± 1.35 | 22.18 ± 5.20 |
| DDP–CON | – 18.10 | + 18.65 | – 0.55 | DDP–CON | – 16.90 | + 9.38 | + 7.52 |

**Table S3**. Lists of antibodies used for western blot analysis

| Antibody | Species | Catalogue number | Company | Dilution | Molecular weight (KD) |
|---|---|---|---|---|---|
| Cdc25A | Rabbit | sc-7157 | Santa | 1:2000 | 70 KD |
| CDK4 | Mouse | #2906 | CST | 1:4000 | 30 KD |
| CDK6 | Rabbit | 3524 | Epitomics | 1:10000 | 37 KD |
| Cyclin D1 | Rabbit | ab134175 | Epitomics | 1:2000 | 36 KD |
| Rb (D20) | Rabbit | #2808 | CST | 1:2000 | 110 KD |
| p-Rb | Rabbit | 8095-1 | Epitomics | 1:2000 | 110 KD |
| PHGDH | Rabbit | A4719-1-AP | Proteintech | 1:1000 | 57 KD |
| PSAT1 | Rabbit | 20180-1-AP | Proteintech | 1:1000 | 40 KD |
| SHMT1 | Rabbit | #12612 | CST | 1:2000 | 50 KD |
| MTHFD1 | Rabbit | 10794-1-AP | Proteintech | 1:1000 | 101 KD |
| CAD | Rabbit | 1709-1 | Epitomics | 1:2000 | 250 KD |
| TS | Rabbit | #9045 | CST | 1:2000 | 30 KD |
| HK2 | Mouse | ab104836 | Abcam | 1:1000 | 102 KD |
| PKM2 | Rabbit | #l53 | CST | 1:2000 | 60 KD |
| LDHA | Mouse | ARH2032 | Antibody Revolution | 1:1000 | 37 KD |
| USP28 | Rabbit | ab110744 | Abcam | 1:2000 | 122 KD |
| c-Myc | Rabbit | 1472-1 | Epitomics | 1:2000 | 57 KD |
| β-actin | Mouse | 66009-1 | Proteintech | 1:5000 | 42 KD |

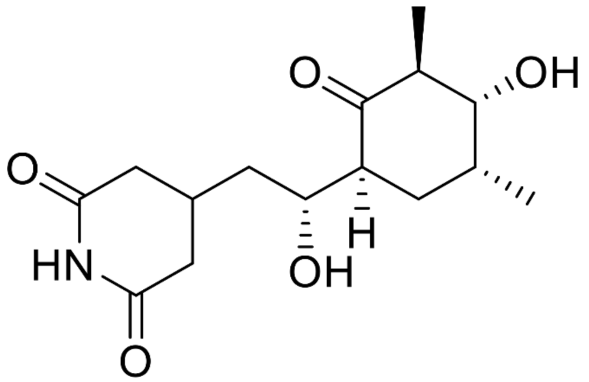


**Figure S1**. Structure of streptoglutarimide H (SGH)

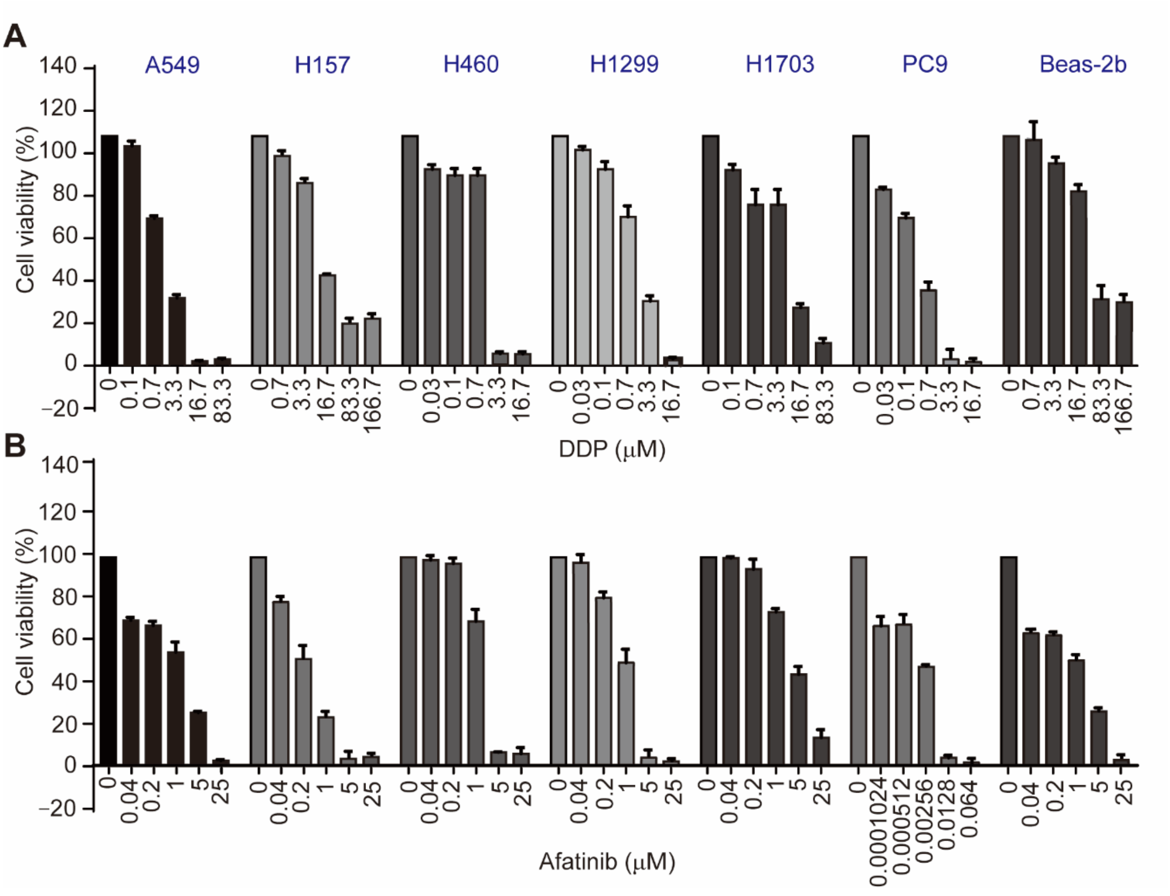


**Figure S2**. Positive control drugs cisplatin (DDP) and afatinib inhibited the proliferation of lung cancer cells and normal lung Beas-2b cells. (**A**) Cell viability of different lung cancer cells and Beas-2b cells treated with different concentrations of DDP for 72 h. (**B**) Cell viability of different lung cancer cells and Beas-2b cells treated with different concentrations of afatinib for 72 h.

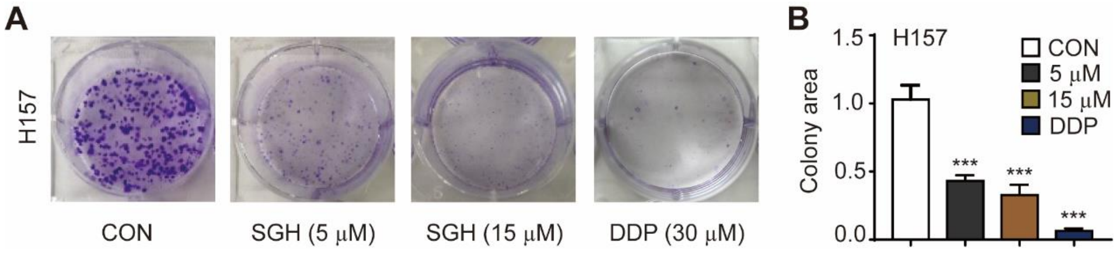


**Figure S3**. Streptoglutarimide H (SGH) and cisplatin (DDP) inhibited the colony formation of lung cancer H157 cells. (**A)** Cell colony of H157 cells treated with SGH (5, 15 μM) or DDP (30 μM) for 2 weeks. **(B)** Quantitative results of the cell colony in Figure S3A. Data are presented as the mean ± SD ($n$ = 3, three independent experiments), ***$p$ < 0.001 (vs. CON) by one-way ANOVA.

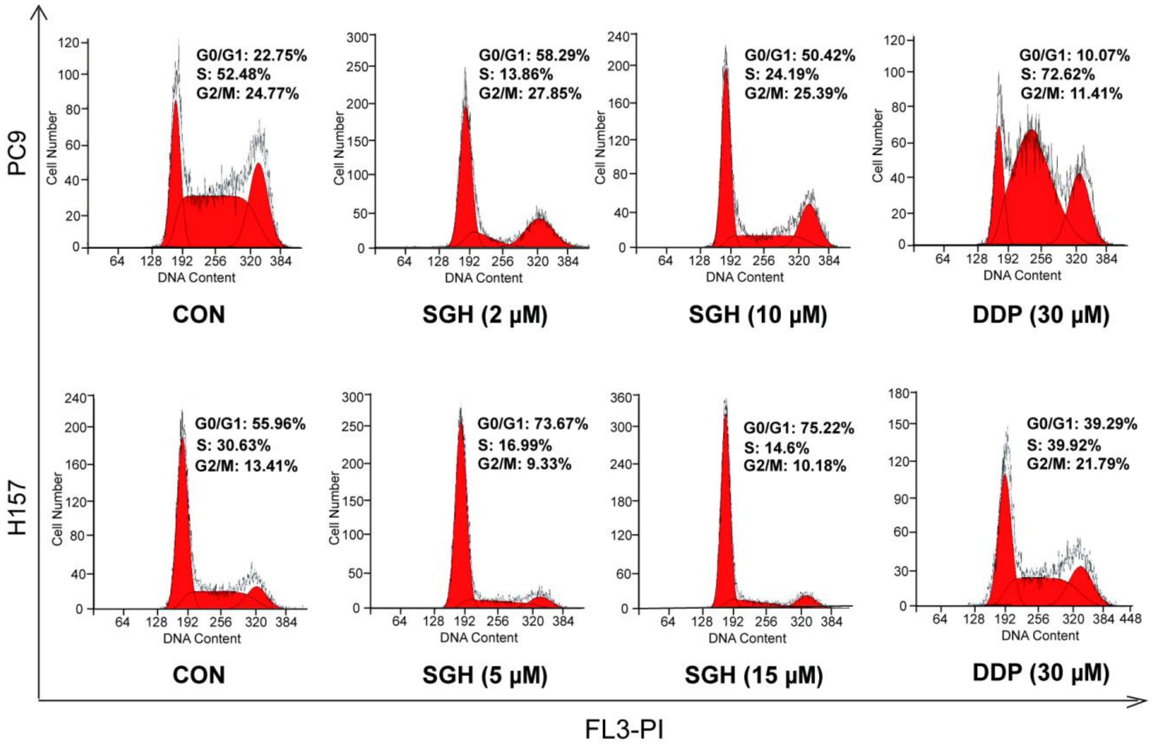


**Figure S4**. Streptoglutarimide H (SGH) and cisplatin (DDP) arrested cell cycle at G0/G1 and S phases, respectively, in lung cancer PC9 and H157 cells. Cells were treated with different concentrations of SGH or DDP (30 μM) for 24 h and percentage of cells at each stage of the cell cycle were shown.

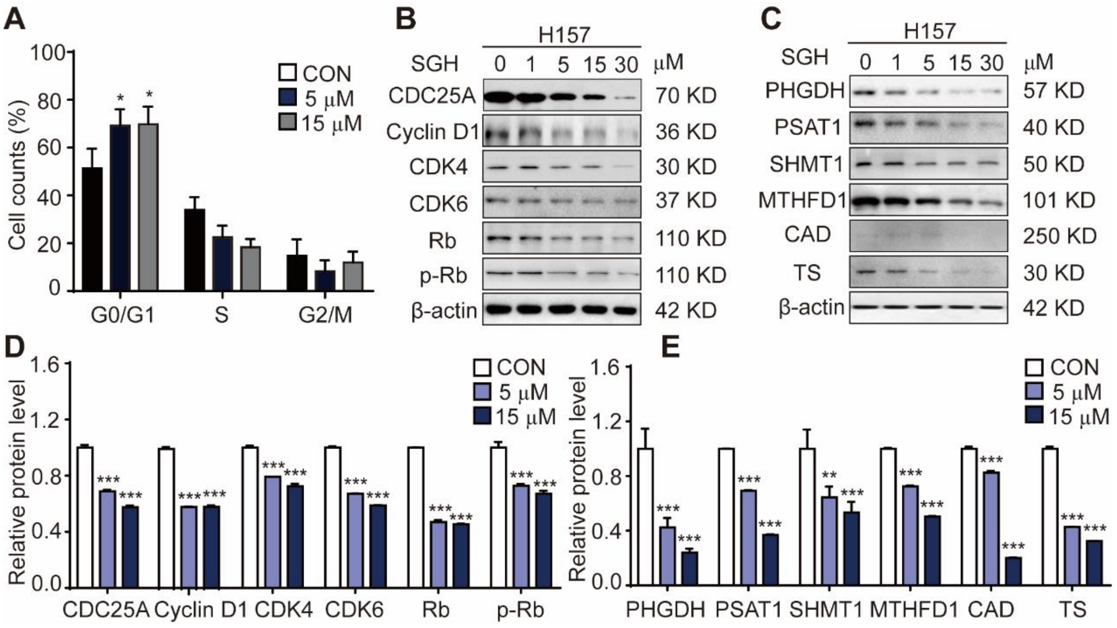


**Figure S5**. Streptoglutarimide H (SGH) arrested cell cycle at G0/G1 phase and downregulated the cell cycle- and nucleotide synthesis-related proteins in lung cancer H157 cells. (**A**) Percentage of cells at each stage of the cell cycle in H157 cells treated with SGH (5, 15 μM) for 24 h. (**B**, **C**) Expressions of the cell cycle- and nucleotide synthesis-related proteins in H157 cells treated with different concentrations of SGH for 24 h. **(D**, **E**) Quantitative results of the protein levels of cell cycle- and nucleotide synthesis-related regulators in Figure S5B and S5C. Data are presented as the mean ± SD ($n$ = 3, three independent experiments), *$p < 0.05$, **$p < 0.01$, ***$p < 0.001$ (vs. CON) by one-way ANOVA.

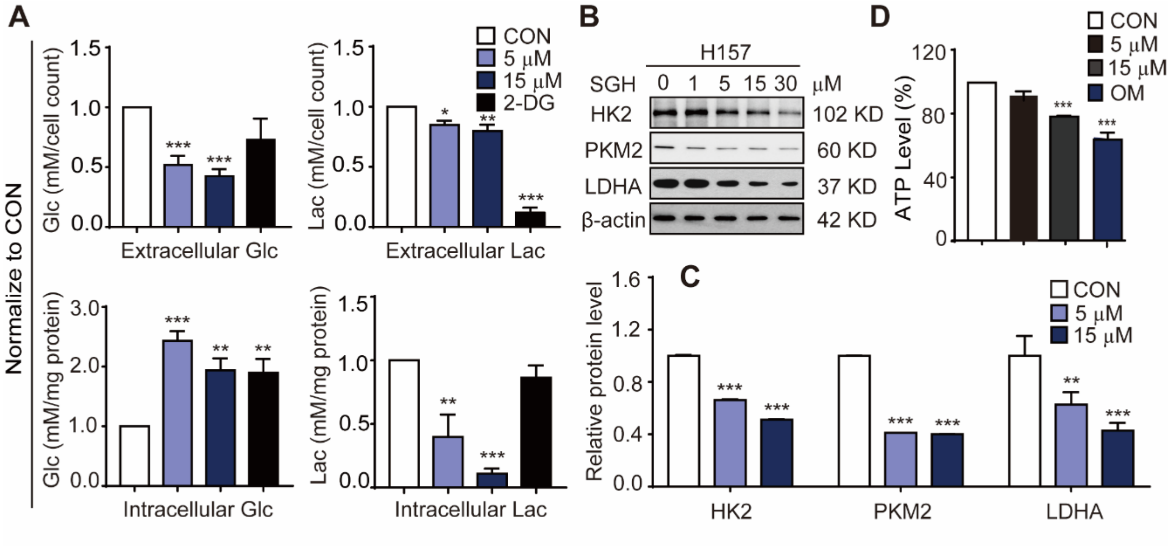


**Figure S6**. Streptoglutarimide H (SGH) inhibited glycolysis in lung cancer H157 cells. (**A**) Extracellular and intracellular glucose (Glc) and lactate (Lac) levels in H157 cells treated with SGH (5, 15 μM) and 2-DG (0.4 mM) for 24 h. (**B**) Levels of glycolytic regulators HK2, PKM2, and LDHA in H157 cells treated with different concentrations of SGH. (**C**) Quantitative results of HK2, PKM2, and LDHA levels in Figure S6B. (**D**) ATP level in H157 cells treated with SGH (5, 15 μM) and oligomycin (OM, 10 μM) for 24 h. Data are presented as the mean ± SD ($n$ = 3, three independent experiments), *$p$ < 0.05, **$p$ < 0.01, ***$p$ < 0.001 (vs. CON) by one-way ANOVA.

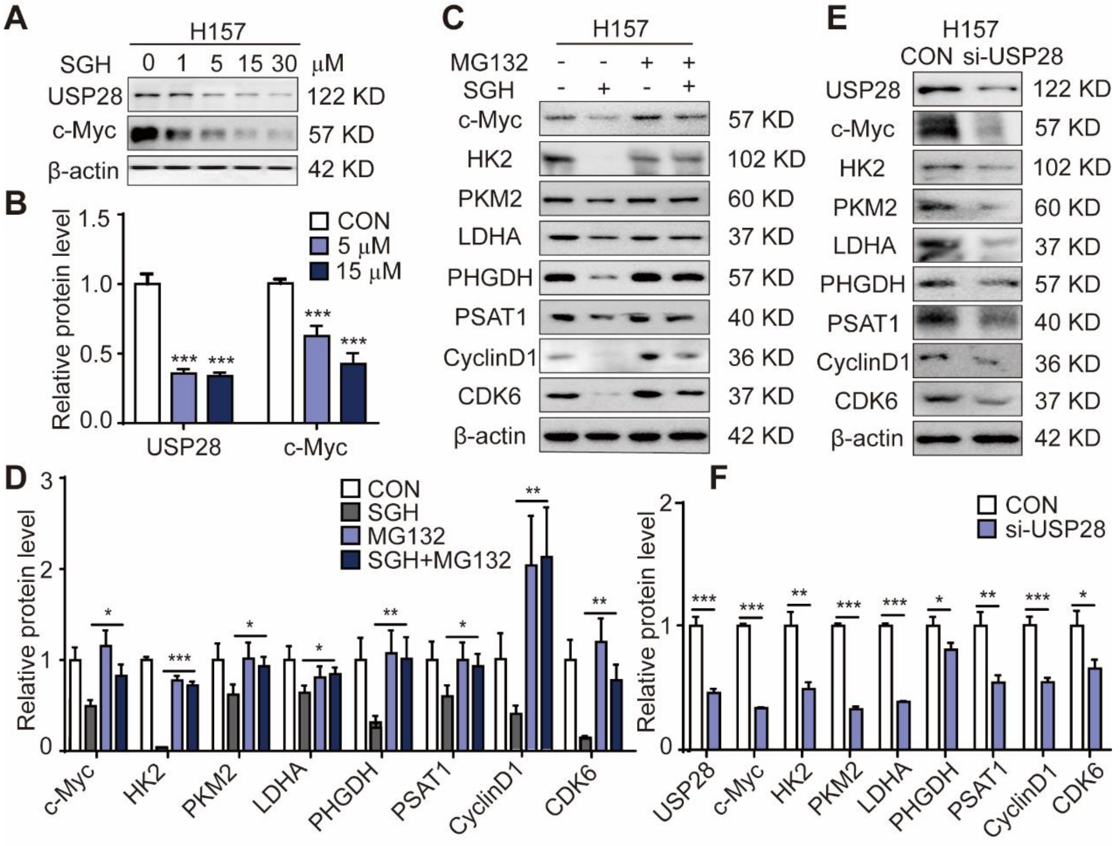


**Figure S7**. Streptoglutarimide H (SGH) downregulated USP28 and c-Myc, proteasome inhibitor MG132 reversed the downregulation of related proteins induced by SGH, and si-USP28 downregulated the cell cycle-, glycolysis-, and nucleotide synthesis-related regulators. (**A**) Protein levels of USP28 and c-Myc in H157 cells treated with different concentrations of SGH for 24 h. (**B**) Quantitative result of USP28 and c-Myc levels in Figure S7A. (**C**) Expressions of c-Myc, HK2, PKM2, LDHA, PHGDH, PSAT1, CDK6, and cyclin D1 in H157 cells pre-incubated with MG132 (10 μM) for 2 h and then SGH (15 μM) for 6 h. (**D**) Quantitative results of the protein levels in Figure S7C. (**E**) Expressions of USP28, c-Myc, HK2, PKM2, LDHA, PHGDH, PSAT1, CDK6, and Cyclin D1 in PC9 cells after the application of 48 h with 20 pmol siRNA. (**F**) Quantitative results of the protein levels in Figure S7E. Data are presented as the mean ± SD (*n* = 3, three independent experiments), $*p < 0.05$, $**p < 0.01$, $***p < 0.001$ (SGH + MG132 vs. SGH or si-USP28 vs. CON) by student′s *t*-test.
